# Negative interpretation bias and emotional responses to positive contexts

**DOI:** 10.1371/journal.pone.0353912

**Published:** 2026-07-30

**Authors:** Emily Gawlik, Karin Coifman

**Affiliations:** Department of Psychological Science, Kent State University, Kent, Ohio, United States of America; Polish Academy of Sciences: Polska Akademia Nauk, POLAND

## Abstract

Negative interpretation bias, the tendency to interpret ambiguous situations as negative or threatening, is associated with reports of intense and more frequent negative affect. However, there is a relative paucity of work seeking to determine whether this cognitive bias might account for individual differences in affective responses to positive content. Given the wealth of benefits associated with positive emotional experiences, such as greater psychological wellbeing and better physical health, this investigation tested the influence of negative interpretation bias on emotion responses to evocative positive film clips as positive emotion-eliciting contexts. Negative interpretation bias was indexed using a novel word matching task. In each investigation, careful consideration of dispositional or trait-level influence on interpretation bias (i.e., dispositional negativity) was considered. Investigations conducted across three samples of college students at a large, Midwestern public university reveal complexity to this question: in one study, a small significant indirect effect of negative interpretation bias on the association between dispositional negativity and self-reported affect following positive emotionally-evocative films was found, while in the two subsequent studies, no significant mediation was found. Certain contextual factors, such as the explicit or discrete nature of the clip presentation as well as more stable tendencies toward positive emotional responsivity and the capacity to self-report emotional experiences, appear to be important. Finally, the novel task was found to be reliable and demonstrated convergent validity with established indicators. Future work should continue to clarify the conditions under which negative interpretation bias may influence responses to positive emotional contexts, given the importance of positive emotionality for lifelong physical and mental health.

## Negative interpretation bias and emotional reactivity to positive contexts

Traditional cognitive models of emotion have identified appraisal, or how a situation is interpreted, as a key driver of emotional experiences [1]. Such processes help explain why two individuals might react to the same situation differently, based on their interpretation of a given context [2]. In the clinical domain, differences in interpretation have been considered one pathway by which some individuals might be more prone to, and have difficulty regulating, frequent and intense experiences of negative emotion [3,4]. These differences may be driven by one or two complimentary pathways, including implicit or automatic attentional shifts [5] that may operate in concert with *later* down-stream interpretations which further enhance the impact of negative or threatening content [6]. Mehu and Scherer [7] emphasized the importance of these types of individual difference factors in contributing to emotion dysregulation and, subsequently, to greater vulnerability to the development of emotional disorders like depression. Indeed, Bylsma and colleagues [8] found that depressed individuals tended to appraise daily life events as more negative and positive events as less pleasant compared to non-depressed counterparts. This pattern emerged not only in individuals currently in the midst of a major depressive episode but also in those who were experiencing subthreshold symptoms of depression, suggesting that emotional appraisals and particularly negatively biased interpretations are meaningfully linked to affective experiences for individuals experiencing clinical *and* non-clinical levels of psychopathology. Importantly, this phenomenon of negatively biased interpretations not only appears to enhance negative emotional responses but may also influence the discounting or downplaying of positive emotional experiences.

### Positive emotion

Deficits in positive emotion, as well as deficits in emotional responses to positive contexts, have great clinical significance. Low levels of positive affect and low responsiveness to reward have been implicated consistently as significant maintaining factors in emotional disorders [9]. There is ample evidence, including meta-analyses looking at longitudinal affective experiences, to suggest that diminished experiences of positive emotion are a risk factor for depression [10]. Additionally, the capacity of individuals to regularly experience positive emotion is also considered a reliable predictor of many desired outcomes. Substantial evidence supports the short-term and long-term benefits of positive emotional experiences for effective coping in stressful situations, cultivating social connections, promoting wellbeing, and even improving physical health outcomes [11,12,13,14]. Hence, a compelling case can be made for delving deeper into the connection between negative biases in attention, interpretation and responsivity to positive emotional experiences.

### Negative interpretation bias

Negative interpretation bias (sometimes alternatively referred to as negative interpretive bias, negative appraisal bias, etc.), or the tendency of an individual to interpret ambiguous circumstances as threatening or negative [15], is a common etiological and maintaining factor in many emotional disorders [16,17]. Interpretation biases often manifest in ambiguous contexts. For example, an individual higher in negative interpretation bias may interpret someone yawning during a presentation they are giving as a sign of boredom or an indication that they are a terrible speaker rather than as something more innocuous, like a sign that this audience member simply did not get enough sleep the night before. However, there is also evidence, particularly among individuals displaying symptoms of social anxiety disorder, of this bias driving more intensely negative interpretations of explicitly positive information. For example, Campbell and colleagues (2009) had individuals with social anxiety and healthy controls make discrete emotion ratings of facial expression stimuli and then also rate the approachability of these faces. Interestingly, socially anxious individuals correctly identified the explicitly happy faces they were shown but rated them as less approachable than non-socially anxious controls. This finding suggests that interpretation biases may influence responses to objectively positive stimuli. Relatedly, several studies have also demonstrated that it may be the tendency to discount *benign* interpretations of ambiguity which ultimately helps to promote more negative interpretations [18,19]. Thus, considering how individuals with interpretation biases make sense of *positive information* may be quite important. Although such biases are commonly present in many psychiatric disorders, it should be noted that individuals who do not exhibit clinical levels of dysregulation can also exhibit these biases [5]. This suggests that the extent to which negative interpretation biases impact emotional experiences has specific implications for clinical populations but is also more broadly relevant.

The body of literature on negative interpretation bias has consistently demonstrated that this bias often predicts increased negative emotional reactivity to stressful events and a limited ability to down-regulate negative emotions [20,17]. There is evidence for negative interpretation bias playing a role in maintaining social anxiety, depression, and posttraumatic stress disorder, as well as being a reliable predictor of symptom severity in these disorders [21,22,23,24,25]. Emerging work has also examined the influence of negative interpretation biases across additional diagnostic categories such as body dysmorphic disorder and generalized anxiety disorder [26]. In examining interpretations of ambiguous vignettes among individuals with various anxiety disorders as well as healthy controls, researchers found notable differences in the content and situations where negative interpretation bias was strongest (e.g., social situations were more salient to the SAD diagnosed group), but generally, negative interpretation bias was present across clinical samples [26]. This suggests an important role for interpretation bias across emotional disorders and that a better understanding of how this cognitive bias works mechanistically may help to make clearer the causal and maintaining processes behind many costly and debilitating psychiatric disorders.

While a fair bit of research exists on how interpretation bias influences individuals’ response to negative content, less focus has been directed toward the impact of interpretation bias on responses to *positive* content [27,15]. However, recent work has begun to remedy this and makes a compelling case for further exploring the link between negative interpretation bias and affective experiences in positive contexts. One such investigation demonstrated that negative interpretation bias, as measured by performance on a variety of tasks assessing both preference for negative words as well as responses to emotional stimuli (e.g., ambiguous faces, ambiguous scenes), was associated with reduced intensity of positive affect in daily reports [28]. Such endeavors benefit from the theory underpinning contemporary cognitive models of emotion, for example, models of emotion regulation flexibility lend credibility to the idea that negative interpretation biases should also impact positive emotion. If attention is focused on negative content and perhaps anticipating negative outcomes, then perhaps the ability to respond to explicit positive cues could be limited [29]. For example, it is well-established that depressed individuals, who often display negative interpretation biases, are less responsive to emotional stimuli broadly, including to positive contexts [30,9]. This may be in part, because of generally inflexible and less responsive patterns of emotional responding, demonstrated compellingly by a failure to discriminate meaningfully across a variety of different emotion stimuli (e.g., objectively sad, amusing, etc.).

Emerging evidence also suggests distinct differences in how depressed individuals exhibiting negative interpretation biases tend to regulate emotion in positive contexts. In addition to being less likely to generate positive emotion when compared to non-depressed counterparts, depressed individuals exhibiting greater negative interpretation bias may tend to dampen responses to positive content [31]. A review conducted by Vanderlind and colleagues indicated that the frequency with which depressed individuals tend to use certain emotion regulation strategies differed notably from the usage of different emotion regulation strategies by non-depressed individuals. More specifically, they found that depressed individuals were more likely to engage in dampening and suppression of positive emotion and less likely to engage in positive rumination or savoring, and they attribute these preferences to biases in attention and interpretation common to depressive disorders. Vanderlind and colleagues also note that these preferences are not only characteristic of depressive episodes but also serve as risk factors for depression and remission of depression symptoms. The persistence of these tendencies outside of depressive episodes suggests relevance for non-clinical populations and implications for the role of negative interpretation bias in onset of depressive symptoms. Work by Everaert and colleagues (2020) [32,33] has probed adjacent questions, assessing whether negative interpretation biases might predict a lower likelihood of positively reappraising situations. One proposed explanation is that it is the rigidity of interpretations, termed interpretation inflexibility, that might best explain the persistence of negative emotion and dampening of positive emotion in ambiguous contexts. Regardless of whether inflexibility can explain this relationship or not, this emergent literature shows promise in better informing the existing knowledge base through identifying ways by which interpretation bias may influence affective experiences.

### The role of dispositional negativity

Seminal work by Barlow and others has demonstrated that underlying dispositional factors linked to temperament can reliably forecast vulnerability for depressive and anxiety disorders [34,35]. Building on these conceptualizations of risk, one useful framework for understanding how negative interpretation bias might fit into the larger picture of affective experience comes from the work of Shackman and colleagues [36]. Integrating lines of research from both psychology and neurobiology, they define this trait-level tendency, called dispositional negativity, as “a propensity to experience and express more frequent, intense, or enduring negative affect” (p. 1276). More specifically, they theorize that dispositional negativity can be thought of as “an extended family of closely related phenotypes,” including narrower, related traits like neuroticism, trait anxiety, behavioral inhibition, and anxiety sensitivity (p. 1277). This corroborates research by Craske and colleagues [37] who also argued for the existence of an underlying trait-level tendency toward negative affect that correlates strongly with these categories of individual difference characteristics.

Mathews and MacLeod [5] have noted that dispositional negativity (also sometimes termed negative affectivity or trait negativity) encompasses a tendency toward negative biases in information processing. This comprises a whole host of different processes, including attention, memory, interpretation, emotional associations, inhibitory control, and more. Deficits in these same processes, in turn, contribute to greater vulnerability, which further emphasizes the importance of considering this as a risk factor for the development of psychopathology. Empirical evidence has also shown that dispositional negativity contributes to vulnerability for the development of anxiety disorders, through mechanisms such as biases toward threat [38]. For example, individuals higher in dispositional negativity often display increased vigilance for threat and attentional biases toward negative and threatening information. These biases not only strain individuals’ cognitive resources and can cause deficits in executive functioning capabilities but also lead to individuals overestimating their likelihood of encountering threats and the intensity or severity of these threats, which is considered a hallmark feature of anxiety disorders [39].

Negative interpretation bias may also potentially be one pathway by which dispositional negativity operates on emotional experiences. Dispositional negativity is associated with less frequent, blunted experiencing of positive affect and reduced sensitivity to reward [40,41]. There is also evidence that dispositional negativity is correlated with lower levels of positive affect and with lower subjective wellbeing [42]. Additionally, dispositional negativity is also associated with negative biases in interpretation [43]. Negative interpretation bias, then, could be considered one mechanism by which individuals higher in dispositional negativity get less out of positive, rewarding contexts. However, it remains unclear exactly how bias itself may drive underlying processes that are associated with diminished reward or insensitivity to positive contexts.

### Current investigation

Previous research has reliably demonstrated the key role negative interpretation biases play in a wide range of emotional disorders and in facilitating the experience of more frequent, intense negative affect. However, far less attention has been afforded to uncovering whether these common biases might exert influence over affective experiences in positive contexts. The current investigation examined how negative interpretation bias, as indexed by a word association task, predicted individuals’ affect after viewing positive emotion-eliciting films. The goal was to test if interpretation bias is a pathway by which dispositional negativity leads to dampened positive affect and elevated negative affect in positive emotional contexts. In addition, this study was also an opportunity to investigate the utility of a novel word association task designed to assess interpretation bias.

To answer these questions, we generated two *a priori* hypotheses. First, negative interpretation bias will be generally associated with dampened emotional responses to positive emotion-evoking stimuli. More specifically, individuals higher in negative interpretation bias will report less positive affect and/or elevated negative affect in response to positive film clips. Second, negative interpretation bias will operate to influence emotional responses to positive contexts when consideration of dispositional negativity is also present. Depending on the experimental framework, this could mean consideration of interpretation bias as a mediator of expected associations between dispositional negativity and elevated negative emotion, or between dispositional negativity and diminished positive emotion. Since this aim is relatively novel, we will also account across our three studies for well-established predictors of positive emotion which may prove to be more influential, such as trait-level positive affect and anhedonia. Individual differences in reward sensitivity play a crucial role in emotion regulatory processes [44,45]. Thus, accounting for any ceiling effects via other key contributors to diminished positive emotion should help to isolate any meaningful independent impact of negative interpretation bias on positive emotion. We conducted three studies in the hopes of testing these proposed relationships. All procedures and methods were approved by the Kent State University Institutional Review Board.

### Transparency and openness

We pre-registered our data analytic plan for Study 2 and our data collection for Study 3. Study 1 was not pre-registered, and sample size was predetermined due to these data having been collected as part of a prior study. SPSS analytical syntax for Study 1 has subsequently been made available on OSF for transparency (https://osf.io/4asgu). Sample sizes for Study 2 and Study 3 were determined based on convenience. All data are available on OSF, and the Negative Interpretation Task (NIT) is also available for download and use (https://osf.io/4asgu/files/osfstorage).

## Study 1

To determine whether negative interpretation bias significantly influences emotional responses to positive contexts, we assessed undergraduate college students’ self-reported affect in response to two positive emotion-eliciting film clips. Negative interpretation bias was measured by a brief, novel word association task, and dispositional negativity was accounted for via validated questionnaires in aggregate. We formulated two hypotheses: 1) Negative interpretation bias will predict dampened emotional responses (greater negative affect, lower positive affect) to positive emotion-evoking stimuli, 2) Negative interpretation bias will *mediate* the relationship between dispositional negativity and positive affect.

## Methods

### Participants

Data were collected January 2020 through May 2020 as part of a larger study [46]. This did include a period of time post-COVID 19 lockdowns (beginning on 3/13/20). Participants were undergraduates at a large, Midwestern public university who were recruited through an online research subject pool. To be eligible for this study, participants were required to be at least 18 years of age and fluent in English.

*N* = 607 undergraduates initiated participation in the study. After excluding individuals who did not complete the questionnaires of interest (n = 39), attempted to participate in the study more than once (n = 11), or who failed two or more of the attention checks and/or gave repetitive responses (n = 50), the total sample available for analysis was n = 507. An independent samples t-test and chi-square test were conducted to determine whether those excluded were meaningfully different from the study sample in terms of demographic characteristics like age, gender, and race. There were no significant differences between the two groups for age. However, there were significant differences for gender, χ(2) = 12.581, *p* = .002, and for the White/Caucasian, χ(1) = 5.976*, p* = .014, and Other ethnicity/race options, χ(1) = 11.020, *p* < .001. Participants excluded tended to be more male, non-white, and more likely to identify as Other when selecting a racial/ethnic identity than the included sample.

This sample had a mean age of 20.01 (SD = 2.95). Eighty-eight percent of participants identified as White/Caucasian, 8.9% as Black/African-American, 4.7% as Asian, 3.6% as Hispanic or Latinx, 2.8% as Other, 1.2% as American Indian/Alaskan Native, and 0.6% as Native Hawaiian/Pacific Islander. The sample was also predominantly female (82%), with 16.6% identifying as male, and 1.0% identifying as Other.

### Procedure

This study was conducted entirely online, using the survey software Qualtrics. After reviewing the study consent form and providing written informed consent, participants completed several questionnaire measures and a novel task designed to assess negative interpretation bias. The task was developed by this research team and modeled after established word association, or homograph-style, tasks [47]. Participants also watched two explicitly positive films previously validated to elicit positive emotional responses [48]. Rigorous testing of these stimuli has established them as being excellent video stimuli that are highly reliable in evoking positive emotion. To confirm that our film clips elicited the discrete positive emotion targets identified in the original stimulus validation papers [48], we performed comprehensive manipulation checks. These tests evaluated the **1) intensity** and **2) discreteness** of affect word ratings, consistent with the original standards set by Gross & Levenson (1995) [49] and the standards still in use today [48,50,51]. Based on assessment of these indices, both clips used in Study 1 were shown to reliably elicit two discrete emotion targets: amusement and happiness. A full description of this process can be found in the Supplemental Materials.

Participants rated their affect prior to questionnaires, after the first film, and after the second film. In addition, participants completed other questionnaires and tasks not relevant to the current investigation. The order in which the two films were presented was randomized, as was the order of all questionnaires and the items (within item sets) of the interpretation bias task.

### Measures

**Affect.** Affect in response to the emotionally evocative film clips was measured via self-report. Participants rated how much they felt a series of emotion words using a Likert-type scale, with 1 indicating “None” and 7 indicating “Strong.” Scores for relief, contentment, happiness, amusement, affection, and interest were averaged to create a positive affect score. Scores for fear, sadness, distress, guilt, anger, and disgust were averaged to create a negative affect score. These particular affect terms have been consistently used in studies of emotional responding and correspond to dominant theories of affective structure (e.g., circumplex models: [52]) and were used in the validation of these films [48]. Scores were calculated after both clip 1 and clip 2, then combined for total PA and NA average across the films (Footnote: Mean PA and NA were also calculated for the individual film clips. The mean PA for clip 1 was M = 3.75, SD = 1.50, while the mean PA for clip 2 was M = 3.02, SD = 1.43. The mean NA for clip 1 was M = 1.40, SD = 0.77, and the mean NA for clip 2 was M = 1.43, SD = 0.80.). The mean positive affect across the sample was M = 3.39, SD = 1.29. The mean negative affect across the sample was M = 1.09, SD = 0.161. Reliability for self-reported positive affect across both video clips was good (Clip 1: α = .879; Clip 2: α = .867). Similarly, reliability for negative affect scores across both video clips was good (Clip 1: α = .860; Clip 2: α = .866).

**Negative interpretation task (NIT).** Negative interpretation bias was assessed using a novel word association task developed by this research team and modeled after other established homograph tasks [Hayes et al., 2010]. In this task, participants are presented with a homograph (i.e., a word that has multiple meanings with the same pronunciation) and asked to select a related word from four options. Two of these choices are related to the prompt word, one being negatively valenced and one being neutral. The other two words are decoys.

Following eight practice trials, participants completed two blocks of 62 trials for a total of 124 trials. Prompt words appear twice, once in the first block and once in the second block. To calculate an interpretation bias score, all correct answers are summed by block, then the total number of negative responses chosen is divided by the total correct responses chosen. Each block is scored, then scores are aggregated across both blocks for a final interpretation bias score. The mean interpretation bias score across both blocks in this sample was M = 0.48, SD = 0.07. Reliability across both blocks of the task was good as indicated by Cronbach’s alpha values (α = .78 and.81). A repeated measures ANOVA indicated a significant decrease in negative bias scores from Block 1 (M = .509, SD = .073) to Block 2 (M = .455, SD = .075), *F*(1, 506) = 97.301, *p* < .001.

**Dispositional negativity.** A trait-level variable representing dispositional negativity was created by aggregating standardized scores from the State-Trait Anxiety Inventory, the Behavioral Inhibition System (BIS) subscale of the Behavioral Inhibition System/Behavioral Activation System Scale, and the Neuroticism subscale of the Big Five Inventory.

The State-Trait Anxiety Inventory (STAI) is a 20-item inventory intended to measure state and trait level anxiety symptoms [53]. We applied the trait version which uses a Likert-type scale ranging from 1 to 4, with scores of 1 indicating that a statement applies “not at all” and 4 indicating that a statement applies “very much so.” Possible scores range from 20 and 80, with higher scores indicating the presence of more severe anxiety symptomology. The mean score in this sample (M = 46.17, SD = 8.35) is considered medium-high based on the recommended cutoff of 40 for anxiety symptoms approaching clinical significance [54]. Internal consistency of the STAI in this sample was very good (α = .917).

The Behavioral Inhibition System (BIS)/Behavioral Activation System (BAS) Scale is a 24-item inventory created to assess motivation, specifically orientations toward approach and avoidance [55]. Scoring corresponds to a Likert-type scale, with scores of 1 meaning that a participant feels a statement is “very true for me” and scores of 4 meaning that a participant feels a statement is “very false for me.” We used only scores on the BIS subscale, which indicates a tendency toward inhibited behavior, or avoidance. The mean score on the BIS in this sample was M = 22.55, SD = 3.52, which is slightly higher than typical scores for individuals of this demographic [56]. Internal consistency of the BIS was decent (α = .782).

The Big Five Inventory is a 44-item inventory that measures five key facets of personality known as the Big Five [57]. Of these five, Neuroticism was used because it characterizes a trait-level propensity toward experiencing frequent and intense negative emotion [58,59]. Internal consistency for the Neuroticism subscale was good (α = .917). The mean for this sample (M = 26.44, SD = 6.48) was consistent with norms for young adults of this age range [60].

Finally, internal consistency of the aggregated dispositional negativity variable itself was quite strong (α = .862).

## Results

### Data analytic plan

In planning the primary analyses, we decided to aggregate positive – or – negative affect across both positive film clips to create a more robust indicator. Mediation analyses were conducted to explicitly test the influence of negative interpretation bias (NIT score) on the association between dispositional negativity (aggregated BIS/Neuroticism/STAI scores) and self-reported positive – or – negative affect after watching the positive films. The Hayes PROCESS macro in IBM SPSS Statistics Version 28.0 was used to run all mediation analyses [61]. Significance was evaluated via bootstrapping (5000 simulations). We planned to first test for mediation without any covariates, for parsimony. Then, if significant mediation was found, we planned (when positive affect was the dependent variable) to add co-occurring negative affect ratings and earlier positive affect ratings as covariates in the model (and vice versa when negative affect was the dependent variable), given likely shared variance between reported negative and positive affect that varies across individuals [62], and the potential for broader mood effects.

### Preliminary analyses

We first confirmed that both positive films succeeded in eliciting positive emotion. A paired-samples t-test indicated that self-reported positive affect (M = 3.39, SD = 1.29) was significantly higher than self-reported negative affect (M = 0.16, SD = 0.007) in response to the positive emotion-evoking films, *t*(506)= 55.964, *p* < .001. The effect size for this difference was large, as was expected, with a Cohen’s *d* of 2.49. We also conducted the more robust manipulation check for both positive film clips, testing for intensity and discreteness of positive affect word ratings, following the process described in the Methods. We confirmed that the clips were operating as expected (see Supplemental Materials).

Next, we ran bi-variate correlations between all key variables (Table 2). Consistent with our first hypothesis, negative interpretation bias was inversely associated with positive affect reported after the first film clip, *r* = −.099, *p* < .001, and the second film clip, *r* = −.186, *p* < .001. In addition, dispositional negativity was positively associated with negative interpretation bias, *r* = .274, *p* < .001, and inversely associated with self-reported positive affect reported after the second film clip, *r* = −.234, *p* < .001. Dispositional negativity was not significantly associated with self-reported positive affect reported after the first film clip, *r* = −.041, *p* = 0.355.

**Table 2:** Correlations across studies.

	Study 1	Study 2	Study 3 (discrete)	Study 3 (mixed)
Dispositional Negativity-NIT	.274**	0.116	.237**	.243**
Dispositional Negativity-Mean PA (Positive Clip)	−.157**	−0.046	0.091	0.024
Dispositional Negativity-Mean NA (Positive Clip)	.318**	.369**	0.047	.163*
NIT-Mean PA (Positive Clip)	−.161**	−0.119	0.002	−0.03
NIT-Mean NA (Positive Clip)	0.071	0.106	−0.019	−0.109

Note: *p < .05. **p < .01. ***p < .001.

### Mediation analyses

**Fig 1:**
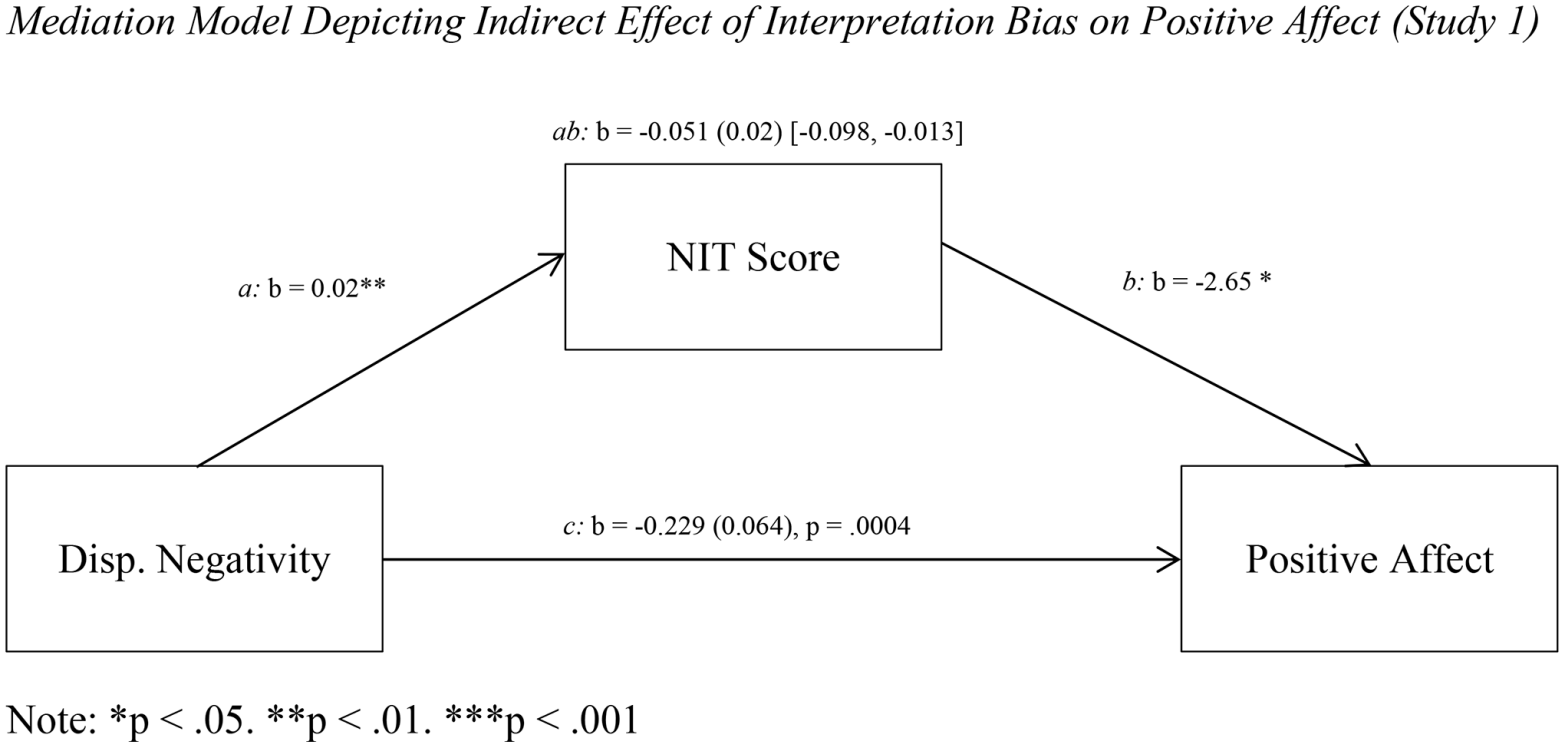
Mediation Model Depicting Indirect Effect of Interpretation Bias on Positive Affect (Study 1).

Our initial mediation model was parsimonious and included no covariates. All paths were significant, as depicted in Fig 1. The a path, which represents the effect of dispositional negativity on negative interpretation bias, was significant, *b* = .02, SE = .003, *p* < .01. The b path, which represents the effect of negative interpretation bias on positive affect, was also significant, *b* = −2.65, SE = .902, *p* = .05. The c’ path, which describes the direct effect of dispositional negativity on positive affect, was significant, *b* = −.178, SE = .066, *p* < .01. Finally, the c path, which represents the total effect of dispositional negativity and interpretation bias on positive affect, was also significant, *b* = −.229, SE = .064*, p* < .01.

We found that in the model with positive affect in response to films as the outcome variable, negative interpretation bias significantly mediated the inverse association between dispositional negativity and PA in response to positive films, R^2^ = .025, *b* = −.051, boot SE = 0.02, 95% CI = [−0.098, −0.013]. In accordance with the data analytic plan for this sample, the model was then run again, with negative affect in response to the positive films included as a covariate. Even when covarying negative affect, significant mediation was still present, R^2^ = .028, *b* = −.052, boot SE = 0.022, 95% CI = [−0.010, −0.015].

Next, earlier reports of positive affect (prior to the completion of questionnaires and viewing of film clips) were included as a covariate. When this earlier positive affect rating was accounted for, mediation was no longer significant, R^2^ = .094, *b* = −.013, boot SE = 0.015, 95% CI = [−0.044, 0.013]

We also ran the same model but with negative affect in response to the positive films as the dependent variable. The a path, which represents the effect of dispositional negativity on negative interpretation bias, was significant, *b* = .020, SE = .003, *p* < .001. The b path, which represents the effect of negative interpretation bias on negative affect, however, was not significant, *b* = −.042, SE = .109, *p* = .700. The c’ path, which describes the direct effect of dispositional negativity on negative affect, was significant, *b* = .059, SE = .008, *p* < .001. Then the c path, which represents the total effect of dispositional negativity and interpretation bias on negative affect, was also significant, *b* = .058, SE = .008, *p* < .001. Hence, mediation analyses did not indicate any significant indirect effect of negative interpretation bias on reports of negative affect in response to the positive films, R^2^ = .101, *b* = −.001, boot SE = 0.02, 95% CI = [−0.005, 0.004], though there was a direct association between dispositional negativity and negative affect as we might expect, *b* = .058, SE = .008, *p* < .001.

## Study 1 discussion

The results of Study 1 were consistent with our hypotheses and suggested a pathway by which interpretation bias might influence the association between dispositional negativity and emotional responses to positive contexts. Indeed, we found correlations as expected between all key variables (i.e., mean PA, dispositional negativity, and negative interpretation bias). As a result, our first hypothesis, which predicted that negative interpretation bias would be associated with dampened emotional responses to positive emotion-evoking stimuli, was supported. We also found support for our second hypothesis, as negative interpretation bias mediated the relationship between dispositional negativity and diminished positive affect. This indirect effect persisted, even when accounting for co-occurring negative affect in response to the positive film.

However, when testing mediation, the indirect effect found was small. Moreover, when earlier positive affect ratings were accounted for in the model, the indirect effect became non-significant. This suggests that the mediation effect might be driven by other factors including general tendencies toward positive affect [63,37]. It should also be noted that the earlier affect ratings did not occur directly before viewing the film clips, which prohibited the calculation of a true reactivity score (i.e., change in positive affect right after watching the film clip compared to positive affect prior to watching the film clip). Thus, these affect measurements might more closely reflect state positive mood rather than positive reactivity. For these reasons, in Study 2 we aimed to replicate our findings and to refine our study design.

## Study 2

With Study 2, we sought to test the same mediation effect and to further investigate the utility of the NIT. In particular, the modifications made to the design of Study 2 allowed us to refine the uniqueness of the positive context by embedding the positive film within a series of films presented in counterbalanced order. Moreover, to account for general tendencies towards low positive affect, participants completed a measure of anhedonic depression, since symptoms of anhedonia are largely consistent with diminished positive affect responses [64]. In addition, we included a task to assess working memory in emotional contexts (RSPAN-E, [65]). Differences in executive cognitive resources could perhaps account for variance in responses to positive emotional stimuli. For example, higher set-shifting ability has previously been shown to predict increased positive emotional responses among high threat-sensitive individuals [66]. Executive functioning, then, could also partly account for the variability in positive affective experiences seen among individuals with dispositional negativity and merits consideration when testing the proposed model. The addition of both anhedonia and emotional working memory capacity measures has further utility in allowing us to account for emotion processing components relevant *early* in information processing, relative to interpretation. We might expect to see, for example, that among individuals who display biases in interpretation, attention, or other relevant cognitive processes, those who display greater emotion-related working memory capacity have an advantage due to this difference in resources and are better able to disengage from those biases. Ultimately, the inclusion of anhedonia and emotional working memory capacity should reveal more about how they intersect with the relationship of interest to this investigation. Finally, to test convergent validity with another, established interpretation bias task, the Emotional Bias Against Disconfirmatory Information (BADE) Task (Everaert et al., 2018) [67] was also administered.

The data-analytic plan for this sample was pre-registered on Open Science Framework (https://osf.io/4asgu). In this pre-registration, we hypothesized, as in Study 1, that negative interpretation bias would be associated with dampened emotional responses (i.e., lower positive affect, elevated negative affect) to the positive emotion-evoking film (the same as in Study 1). Furthermore, we expected that negative interpretation bias could mediate the relationship between dispositional negativity and emotional responses to this positive film context.

## Methods

### Participants

Data were collected September 2022 through November 2022. Participants were undergraduates at a large, Midwestern university, recruited through the department subject pool. Eligibility criteria were the same as in Study 1.

Data were obtained for *N* = 203 participants. Participants were excluded if they 1) did not complete the study or tried to complete the study more than once (n = 8), 2) failed any of the five total attention checks in the study (n = 50), 3) responded to survey items in an overly repetitive manner (n = 2), or 4) scored two standard deviations above or below the mean on the NIT (n = 5). Thus, the final sample consisted of n = 138 participants. Independent samples t-tests were conducted to determine whether those excluded were meaningfully different from the study sample in terms of demographic characteristics like age, gender, and race. There were no significant differences between the two groups for age or for gender, χ(3) = 2.655, *p* = .448. There were, however, significant differences for race, χ(5) = 14.602, *p* = .012. Specifically, participants who were excluded were more likely to identify as Black/African American or Middle Eastern than those included.

This sample had a mean age of 20.24 (SD = 4.90). Eighty-two percent of participants identified as White/Caucasian (n = 113), 4.3% as Black/African-American (n = 6), 4.3% as Asian or Pacific Islander (n = 6), 2.2% as Hispanic or Latinx (n = 3), 0.7% as American Indian/Alaskan Native (n = 1), and 6.4% chose not to respond (n = 9). The sample was also predominantly female, with 79.7% of participants identifying as such (n = 110), 15.2% as male (n = 21), and 5.1% as non-binary/third gender (n = 7).

### Procedure

Since additional activities were added to Study 2, participation was split into two one hour-long online sessions to reduce burden on the participant. All participants were required to complete both sessions within one week. After reviewing the study consent form and providing written informed consent, participants reported demographics and questionnaires assessing relevant trait-level characteristics and psychological symptoms. Just as in Study 1, participants were asked to complete the NIT. They were also shown validated [48] emotionally-evocative film clips, which included one negative emotion eliciting film clip, one positive emotion eliciting film clip (same as Study 1, Clip A), and one neutral film clip. Presentation of the films was counterbalanced, such that *all* participants were shown the neutral film first but whether they would next see the positive film or next see the negative film was random. Participants provided affect ratings at the beginning of the survey, before the films, and then after each film. To confirm that our film clips elicited the discrete positive emotion targets identified in the original stimulus validation papers [48], we once again performed comprehensive manipulation checks consistent with the standards set by Gross & Levenson (1995) [49]. The film clip again reliably elicited amusement and happiness in Study 2. A full description of this process can be found in the Supplemental Materials.

### Measures

**Dispositional negativity.** As in Study 1, dispositional negativity was represented by an aggregated variable, derived from scores on neuroticism, behavioral inhibition, and trait anxiety measures. Scores were aggregated as in Study 1, and means (see Table 1) were consistent with prior research and means found in the Study 1 sample [54,56,60]. Internal consistency of the aggregated variable was strong (α = .819).

**Table 1:** Descriptive statistics for key variables.

	Study 1	Study 2	Study 3 (discrete)	Study 3 (mixed)
STAI	46.17(8.35)	49.91(8.21)	49.13(9.87)	47.80(9.95)
BIS	22.55(3.52)	23.34(3.38)	22.97(3.71)	23.13(3.59)
BFI/BFI-SF	26.44(6.48)	27.62(6.60)	20.21(5.43)	19.34(5.13)
NIT	.48(0.07)	.48(.07)	.48(.07)	.48(.06)
PA (funny cats)	3.75(1.50)	3.77(1.43)	4.00(1.56)	2.93(1.59)
NA (funny cats)	1.40(0.77)	1.31(0.59)	1.21(.49)	2.00(1.13)

Note: Values follow M(SD) format; BFI was used to measure neuroticism in Study 1 and 2, while BFI-SF was used in Study 3.

**Negative interpretation bias**. Once again, negative interpretation bias was measured using the NIT. The same scoring rules were used for this sample as in Study 1. When cleaning the task data for this sample, n = 3 participants were excluded due to having scores that were three standard deviations above or below the sample’s mean interpretation bias score. The mean NIT score for this sample (M = 0.48, SD = 0.07) resembled the distribution of scores found in Study 1. Reliability of both blocks was acceptable (α = .732 and.658). A repeated measures ANOVA indicated a significant decrease in negative bias scores from Block 1 (M = .503, SD = .057) to Block 2 (M = .455, SD = .064), though this was expected, *F*(1, 137) = 97.301, *p* < .001.

An additional measure of negative interpretation bias, the Emotional Bias Against Disconfirmatory Information (Emotional BADE) Task, was also administered. In this task, participants are presented with a series of vignettes that describe ambiguous scenarios, each followed by a series of additional statements that give further context to the scenario [Everaert et al., 2018]. After receiving each statement, participants rate how plausible all four interpretations of the scenario are. One of these interpretations is absurd (never seems plausible), some are “lure” interpretations (plausible at first but are less plausible after each contextual statement), and some are true interpretations (less plausible at first but more plausible once additional information about the situation is learned through each statement). This task assesses both interpretation bias as well as interpretation flexibility. Scoring for this task followed set recommendations [Everaert et al., 2018]. In the case of the negative interpretation bias index of the Emotional BADE Task, this meant averaging the plausibility ratings for “lure” interpretations across all vignettes. The mean of the negative interpretation bias index for all scenario types in this sample (M = 9.29, SD = 1.50) was consistent with prior research [67].

**Working memory.** Emotion-linked working memory capacity was measured using an emotional ‘reading span’ task (RSPAN-E, [65]). In this task, participants are presented with a logical or nonsensical sentence with a negative affective valence and an unrelated neutral word. Participants read the sentence and then report if the sentence makes sense, then read the unrelated neutral word. At the end of a set, participants are asked to recall as many words as they can remember, in serial order. Scoring follows a partial-credit system, where participants receive points per set based on how many words they remembered. A final score is then calculated by averaging performance across all sets. For this task, responses are excluded if participants do not accurately identify the sentences as logical or illogical. To clean the task data, we first excluded participants who showed clear non-compliance with the task instructions through repeated non-responses. Based on this attention check, n = 38 participants were excluded. Next, individuals whose processing index scores (i.e., proportion of sentences identified as logical or illogical) were two standard deviations below the sample mean were excluded, keeping with precedent for cleaning RSPAN data [65]. Based on this cleaning rule, n = 2 participants were excluded. This resulted in *N* = 78 participants having reliable RSPAN scores. The distribution of their RSPAN scores (M = .57, SD = .17) was consistent with prior research in college students [65,68]. Processing index scores were also typical of college student samples (M = .69, SD = .12).

**Affect.** Responses to each film were measured via self-report as in Study 1. Participants again rated how much they felt a series of emotion words using a Likert-type scale, with 1 indicating “None” and 7 indicating “Strong,” and positive affect and negative affect scores were calculated using the same sets of affect words. For Study 2, affect ratings collected immediately prior to the positive film clip made it possible to derive change scores, which may more accurately assess emotional reactivity. The mean positive affect change score for the positive film clip was M = .574, SD = 1.40, while the mean negative affect change score following the positive film clip was M = −.814, SD = 0.916. Reliability of these affect ratings were comparable to those found for affect ratings in Study 1.

**Anhedonic depression.** Participants completed the Anhedonia subscale of the Mini-Mood and Anxiety Questionnaire (Mini-MASQ; [69]). The mean anhedonia score for this sample (M = 23.20, SD = 7.37) was comparable to norms from another sample of Midwestern college students [69]. Reliability of the measure was also good (α = .798).

## Results

### Data analytic plan

The data-analytic plan for Study 2 was pre-registered on Open Science Framework (https://osf.io/4asgu). Mediation analyses were conducted to examine the effect of negative interpretation bias (NIT score) on the relationship of dispositional negativity (aggregated BIS/Neuroticism/STAI scores) and emotional responses to the positive film. In our primary model, we planned to first test for mediation with no covariates, in keeping with what was done in Study 1. Then, if significant mediation was found, we planned to consider negative affect as a covariate in the model. As an additional test of rigor, we would run this model again with anhedonic depression as a covariate. Following this, we planned to test the incremental validity of the NIT by adding the RSPAN to our model and then adding the BADE. We did not plan to analyze responses to the negative film, as it was included with the intent of making the positive film as a context, more explicit.

### Preliminary analyses

We first checked to ensure that the positive film succeeded in eliciting positive emotion as expected. Paired-samples t-tests indicated that self-reported positive affect (M = 3.77, SD = 1.43) was significantly higher than self-reported negative affect (M = 1.31, SD = 0.59) after viewing the positive emotion-evoking film, *t*(137)= 17.89, *p* < .001. Self-reported positive affect after viewing the positive film was also greater than positive affect after viewing the negative film, *t*(137)= 14.86, *p* < .001. Nega*t*ive affect after viewing the negative film was also greater than negative affect after viewing the positive film, *t*(137)= 13.896, *p* < .001.

We next ran bi-variate correlations between all key variables (see Table 2). Unlike in Study 1, NIT was not significantly correlated with either mean PA change in response to the positive film, *r* = −.006, *p* = .947, nor with mean NA change in response to the positive film, *r*(138) = −.075, *p* = .380. In addition, dispositional negativity was not significantly correlated with negative interpretation bias (as measured by the NIT), *r* = .116, *p* = .176. However, dispositional negativity *was* significantly associated with mean PA change in response to the positive film, *r* = −.208, *p* = .014 and inversely associated with mean NA change in response to the positive film, *r* = .455, *p* < .001, like in Study 1.

Anhedonic depression was significantly correlated with dispositional negativity, *r* = .369, *p* < .001, and negative interpretation bias, *r*(138) =.185, *p* = .05. Anhedonic depression was also significantly correlated with mean NA change in response to the positive film, *r* = .298, *p* < .001, though not significantly correlated with mean PA change.

The Emotional BADE Task (Negative Interpretation Bias index) was significantly correlated with negative interpretation bias as measured by the NIT, *r* = .226, *p* < .05, providing evidence for convergent validity with the NIT. Emotional BADE scores were not significantly correlated with any of the affect response variables but were associated with dispositional negativity, *r* = .234, *p* < .001. Finally, RSPAN-E scores were not significantly correlated with any variable.

### Mediation analyses

**Fig 2:**
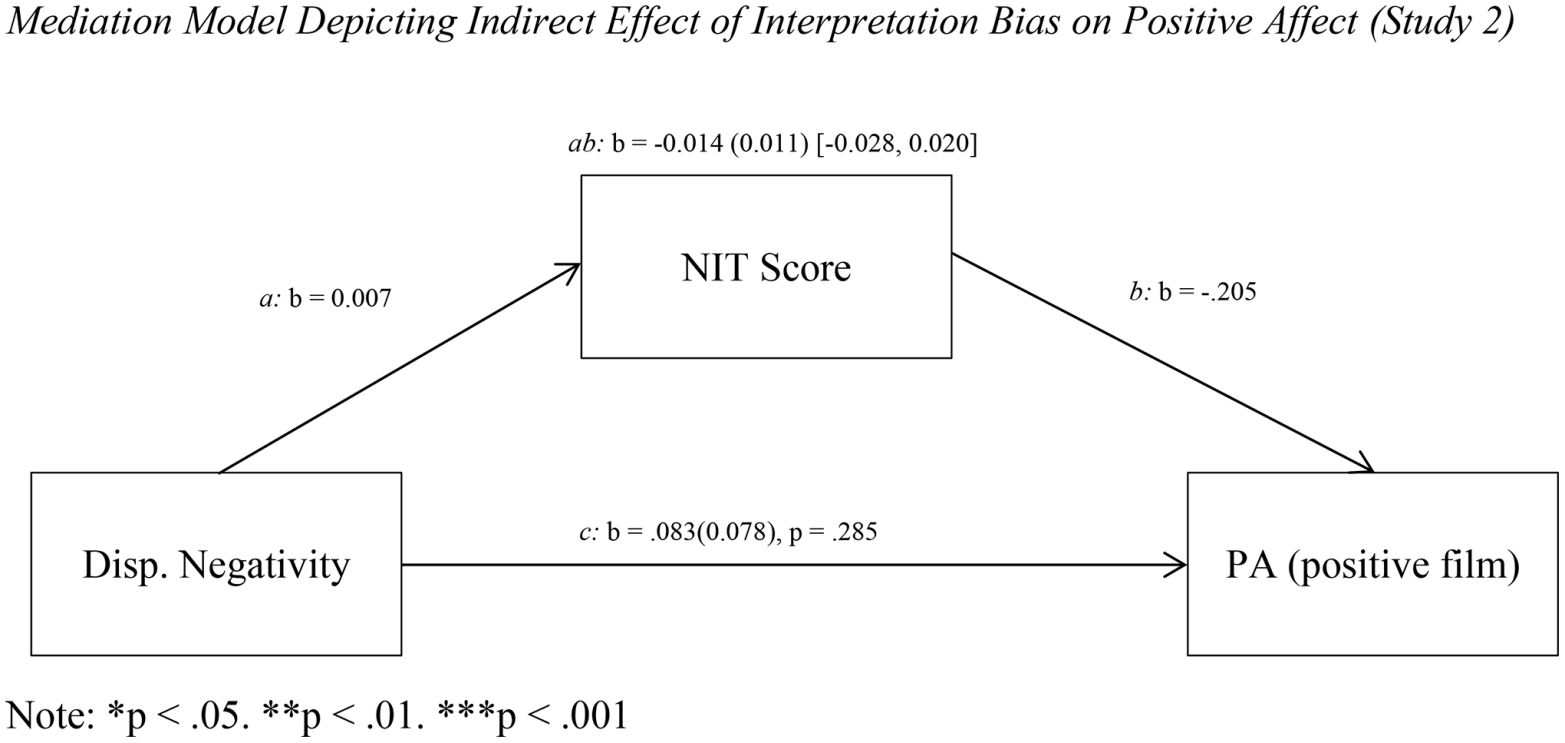
Mediation Model Depicting Indirect Effect of Interpretation Bias on Positive Affect (Study 2).

Our initial mediation model was parsimonious and included no covariates. All paths of this mediation model are depicted in Fig 2. The a path, which represents the effect of dispositional negativity on negative interpretation bias, was non-significant, *b* = .007, SE = .005, *p* = .176. The b path, which represents the effect of negative interpretation bias on PA change score, was also non-significant, *b* = −.817, SE = 2.321, *p* = .726. The c’ path, which describes the direct effect of dispositional negativity on PA change score, was significant, *b* = −.338, SE = .136, *p* = .014. Hence, we found that negative interpretation bias did not significantly mediate the inverse association between dispositional negativity and PA change score, R^2^ = .043, *b* = −.006, boot SE = 0.021, 95% CI = [−0.058, 0.033], though a significant direct effect was exhibited. Since mediation was not present for positive affect responses, we did not go on to run the model again covarying mean NA.

Next, we ran a similar mediation model, with NA change score as the outcome variable. The a path, which represents the effect of dispositional negativity on negative interpretation bias, was non-significant, *b* = .007, SE = .005, *p* = .176. The b path, which represents the effect of negative interpretation bias on NA change score, was also non-significant, *b* = .421, SE = 1.378, *p* = .760. However, the c’ path, which describes the direct effect of dispositional negativity on NA change score, was significant, *b* = .473, SE = .081, *p* < .001. Hence, we found that negative interpretation bias did not significantly mediate the inverse association between dispositional negativity and NA change score either, R^2^ = .013, *b* = .003, boot SE = 0.013, 95% CI = [−0.032, 0.023], though a significant direct effect was exhibited.

### Exploratory analyses

As detailed in the pre-registration (https://osf.io/4asgu), we also conducted planned exploratory analyses. First, we replaced the NIT with the Emotional BADE Task in the model to test whether associations similar to those seen in Study 1 would be found when using another, established measure of interpretation bias.

In our primary model, the a path, which represents the effect of dispositional negativity on BADE scores, was significant, *b* = .408, SE = .156, *p* < .001. The b path, which represents the effect of BADE scores on PA change score, was non-significant, *b* = .117, SE = .088, *p* = .105. The c’ path, which describes the direct effect of dispositional negativity on PA change score, was not significant either, *b* = −.034, SE = .154, *p* = .828. Thus, BADE negative interpretation scores did not significantly mediate the inverse association between dispositional negativity and PA change score, R^2^ = .055, *b* = 0.048, boot SE = 0.041, 95% CI = [−0.022, 0.141].

We ran our second mediation model, with NA change score as the outcome variable. The a path, which represents the effect of dispositional negativity on BADE scores, was significant, *b* = .408, SE = .156, *p* < .001. The b path, which represents the effect of BADE scores on NA change score, was non-significant, *b* = .001, SE = .030, *p* = .988. However, as before, the c’ path, which describes the direct effect of dispositional negativity on NA change score, was significant, *b* = .185, SE = .052, *p* < .001. Hence, we found that BADE scores did not significantly mediate the inverse association between dispositional negativity and NA change score either, R^2^ = .055, *b* = .0002, boot SE = 0.012, 95% CI = [−0.023, 0.026], though the same significant direct effect was exhibited.

Alternative explanations for the primary mediation effect detected in Study 1 were also tested in this data collection. We considered whether this indirect effect could instead be explained by variability in working memory capacity or generalized low levels of positive affect. As such, we planned to insert both variables into the mediation models described above alongside the NIT. While we did initially run these models, despite not finding a significant indirect effect of the NIT in our primary analyses, we did not find that either model accounting for working memory capacity or anhedonic depression showed significant mediation. Thus, these models are presented in the Supplemental Materials.

## Study 2 discussion

With Study 2, we aimed to replicate the mediation effect found in Study 1 and to address Study 1’s design limitations. We intended to conduct a more robust test of negative interpretation bias’s influence on emotional reactivity to positive contexts. We hypothesized that negative interpretation bias would be associated with dampened positive affect and elevated negative affect in response to a positive film, and that negative interpretation bias would also mediate the relationship between dispositional negativity and emotional responses to the positive film. However, notably in Study 2, we did not find significant associations between dispositional negativity and negative interpretation bias or between NIT scores and PA or NA, as was expected in the preliminary analyses. Additionally, significant mediation was not found in the primary models that were tested. Thus, neither of our hypotheses were supported, and the effect found in Study 1 was not replicated with this sample.

Interestingly, anhedonic depression was significantly correlated with a number of key variables, including dispositional negativity and negative interpretation (as measured by the NIT and the Emotional BADE Task), as well as with mean PA and NA in response to the positive film. We also found that the NIT and Emotional BADE were significantly correlated, which demonstrates convergent validity but also suggests that the tasks are not redundant with one another.

In considering the lack of replication of mediation in Study 2, we next aimed to manipulate one more factor in our investigation to determine how and/or when we might detect these associations. Specifically, we focused on manipulating the degree of ambiguity present in each evocative context. Prior research has suggested that interpretation biases operate most prominently in contexts with ambiguity. In Study 2, we attempted to make the positive film more explicitly positive by embedding it within a series of other evocative films. This could potentially account for the replication failing. Hence, in Study 3, we focused on addressing this dimension of our study design in the hopes of reconciling our mixed findings from Study 1 and 2.

## Study 3

In Study 3, we expanded upon the previous two studies by now explicitly manipulating the film contexts in which we measured affective responses. Specifically, we performed a direct test of our model in an ambiguous positive film context versus an explicit positive film context. Prior work, both theoretical and experimental, has established that negative interpretation biases emerge strongest in the presence of ambiguity [5,15; 22]. To achieve this, we employed a between-groups experimental design, where participants were randomized to view either two discrete emotionally evocative film clips or two mixed (or ambiguous) emotionally evocative film clips.

The data-analytic plan for Study 3 was pre-registered on Open Science Framework (https://osf.io/7gdhw). We hypothesized that higher negative interpretation bias, measured using the NIT, would predict lower positive affect and elevated negative affect responses in the group viewing *mixed* film clips relative to the group viewing *discrete* film clips. We then planned to confirm these effects were present even when considering dispositional negativity in our models.

## Methods

### Participants

Data were collected November 2023 through December 2023. Recruitment and eligibility criteria were the same as in Studies 1 and 2. Data were obtained for *N* = 377 participants. Participants were then excluded if they 1) did not complete the study (n = 23) or 2) failed any of the seven total attention checks in the study (n = 60). Following these exclusions, the final sample consisted of *N* = 294 participants. Independent samples t-tests and chi-square tests were conducted to ascertain whether those excluded were meaningfully different from the study sample on demographic characteristics such as age, gender, and race. There were no significant differences between the two groups for age, *t*(363) =.953, *p* = .171, or for gender, χ = 4.298, *p* = .231. There were, however, significant differences for race. Specifically, excluded participants were more likely to identify as Black/African American, χ = 17.475, *p* = .001, or White/Caucasian, χ = 21.706, *p* = .001, than the included sample.

This sample had a mean age of 19.98 (SD = 3.53). 85.0% of participants identified as White/Caucasian (n = 252), 8.8% as Black/African-American (n = 26), 5.1% as Asian or Pacific Islander (n = 15), 4.4% as Hispanic or Latinx (N = 13), 0.7% as American Indian/Alaskan Native (n = 2), and 0.7% as Other (n = 2). The sample was also predominantly female, with 77.2% of participants identifying as such (n = 227), 19.7% as male (n = 58), and 3.0% as non-binary/third gender (n = 9).

### Procedure

Again, all activities were completed online via the survey platform Qualtrics. After reviewing the study consent form and providing written informed consent, participants completed several questionnaire measures of individual differences in trait anxiety, behavioral inhibition/activation, anhedonic depression symptoms, and Big Five personality traits, as well as some additional psychological measures not relevant to this investigation. As in Studies 1 and 2, participants also completed the NIT, to assess negative interpretation bias. After the NIT, participants were randomized to watch one of two possible sets of emotionally-evocative videos. The “discrete” set consisted of a negative film (a scene from the movie Trainspotting) and a positive film (a compilation video of cats doing amusing things, used in Study 1 and 2). The “mixed” set consisted of the same negative film clip but with a laugh track overlaid and the same positive film clip but with negative audio (i.e., vomiting sounds) overlaid, with the intent of rendering the film clips emotional tone more ambiguous. Following each video, participants provided affect ratings for the same list of emotion words used in Study 1 and Study 2.

### Measures

**Dispositional negativity.** We calculated a dispositional negativity variable by aggregating the same measures as in Study 1 and Study 2. Scores on the individual measures and Cronbach’s alpha estimates of reliability were similar to those found in the prior two studies (BFI-N-SF: M = 19.77, SD = 5.29, α = .829; BIS subscale: M = 23.05, SD = 3.64, α = .809; STAI: M = 48.45, SD = 9.92, α = .895). The mean dispositional negativity score for this sample was also consistent with scores in our other two samples (M = 0.04, SD = 0.88), and its own internal consistency was decent (α = .747).

**Negative interpretation bias.** As in Studies 1 and 2, we measured negative interpretation bias using the NIT. We used the same scoring rules as in both prior studies. No participants were excluded due to having scores three standard deviations above or below the sample’s mean interpretation bias score. The NIT scores for this sample (M = 0.48, SD = 0.003) resembled the distribution of scores found in the Study 1 and 2 samples. Reliability of both blocks of the task was good (α = .793 and.692). A repeated measures ANOVA indicated a significant decrease in negative bias scores from Block 1 (M = .505, SD = .075) to Block 2 (M = .452, SD = .070), though this was expected, F(1, 293) = 182.00, p < .001.

**Affect.** Participants provided affect ratings prior to viewing any video clips, after viewing the first video clip, and after viewing the second video clip. Negative and positive affect scores were derived as in Studies 1 and 2. Reliability of affect ratings ranged from good to acceptable (Cats Mixed PA α = .890, Cats Mixed NA α = .799, Cats Discrete PA α = .910, Cats Discrete NA α = .779), and the distribution of affect ratings was in line with expectations. Again, change scores were calculated to more closely assess reactivity to emotional stimuli using affect ratings prior to viewing the film clips and directly after viewing the clips.

## Results

### Data analytic plan

The data-analytic plan for Study 3 was pre-registered on Open Science Framework (https://osf.io/7gdhw). Our primary analysis consisted of two separate tests via OLS regression where the dependent variable was self-reported affect in response to the positive film clip (discrete or with mixed audio). For both tests, group assignment to film type (discrete v. mixed) served as the moderator. The primary predictor was negative interpretation bias, as measured by the NIT, with the interaction term entered in the second step. We did not consider age or sex as covariates as there was no evidence of differences between groups, suggesting random assignment worked as intended. As in Study 2, we did not plan to analyze responses to the negative clip (discrete or mixed) since it was included simply to provide an explicit contrast to the positive film/context.

### Preliminary analyses

We first tested for equivalence of randomized groups. We found no significant differences in dispositional negativity (*p* = .205) nor negative interpretation bias (*p* = .353) between the two groups, and there were no significant differences in gender (*p* = .576) nor race (all *p* > .05) between groups. There was a significant difference between groups in age, such that participants in the discrete films group were slightly older on average, *t*(289) = 1.497, *p* = .041.

We confirmed the film effectiveness with paired-samples t-tests indicating that self-reported positive affect (M = 3.46, SD = 1.66) was significantly higher than self-reported negative affect (M = 1.61, SD = .96) after viewing either the discrete or mixed positive emotion-evoking film, *t*(293) = 14.47, *p* < .001. The effect size for this difference was large, with a Cohen’s *d* of 2.18. We also found that self-reported negative affect (M = 2.72, SD = 1.08) was significantly higher than self-reported positive affect (M = 1.75, SD = .87) after viewing the discrete or mixed negative emotion-evoking film, *t*(293) = 11.86, *p* < .001. The effec*t* size for this difference was more modest, with a Cohen’s *d* of.69.

Additionally, to test whether the incongruent audio overlay resulted in the mixed film becoming explicitly ambiguous, we conducted a manipulation check using within-subjects contrasts of the discrete affect words “amusement” and “disgust.” As expected, we found that both amusement (M(SD) = 4.54(1.81)) and disgust (M(SD) = 3.41(2.38)) were elicited at high intensity. Amusement ratings were significantly higher than ratings on any other positive emotion word besides happiness, while amusement and disgust were elicited at levels that were not significantly different, F(1, 293) =.559, p = .455. Next, we conducted zero-order correlations between all key variables, including dispositional negativity, negative interpretation bias, and affect. As in Study 1, we found that dispositional negativity and NIT score were significantly correlated, *r* = .243, *p* < .001. Dispositional negativity was also significantly correlated with anhedonic depression symptoms, *r* = .632, *p* < .001, and with PA responses to the mixed film clip, r = −.113, p = .023. NIT scores were not significantly associated with NA or PA responses to either film clip, though NIT scores were significantly associated with anhedonic depression symptoms, *r* = .244, *p* < .001.

### Primary analyses

We used model number 7 in PROCESS to run two moderated-mediation models, predicting two possible outcomes–1) PA responses to the positive film and 2) NA responses to the positive film. Specifically, group assignment to mixed versus discrete clips was the moderator and we expected that group would moderate the mediational action of NIT score on the association between dispositional negativity and affective responses as dependent variable.

In our model predicting PA change scores, while there was a significant direct effect of dispositional negativity on PA responses (*b* = .180, SE = .081, t = 2.215, p = .027), condition was not found to moderate the indirect effect. The overall moderated mediation model was not supported, according to the index of moderated mediation (Condition = .002 (95% CI = −.013,.022). Since zero was contained within the confidence interval, we did not find evidence for a significant moderating effect of condition on dispositional negativity on the indirect effect via NIT.

**Fig 3:**
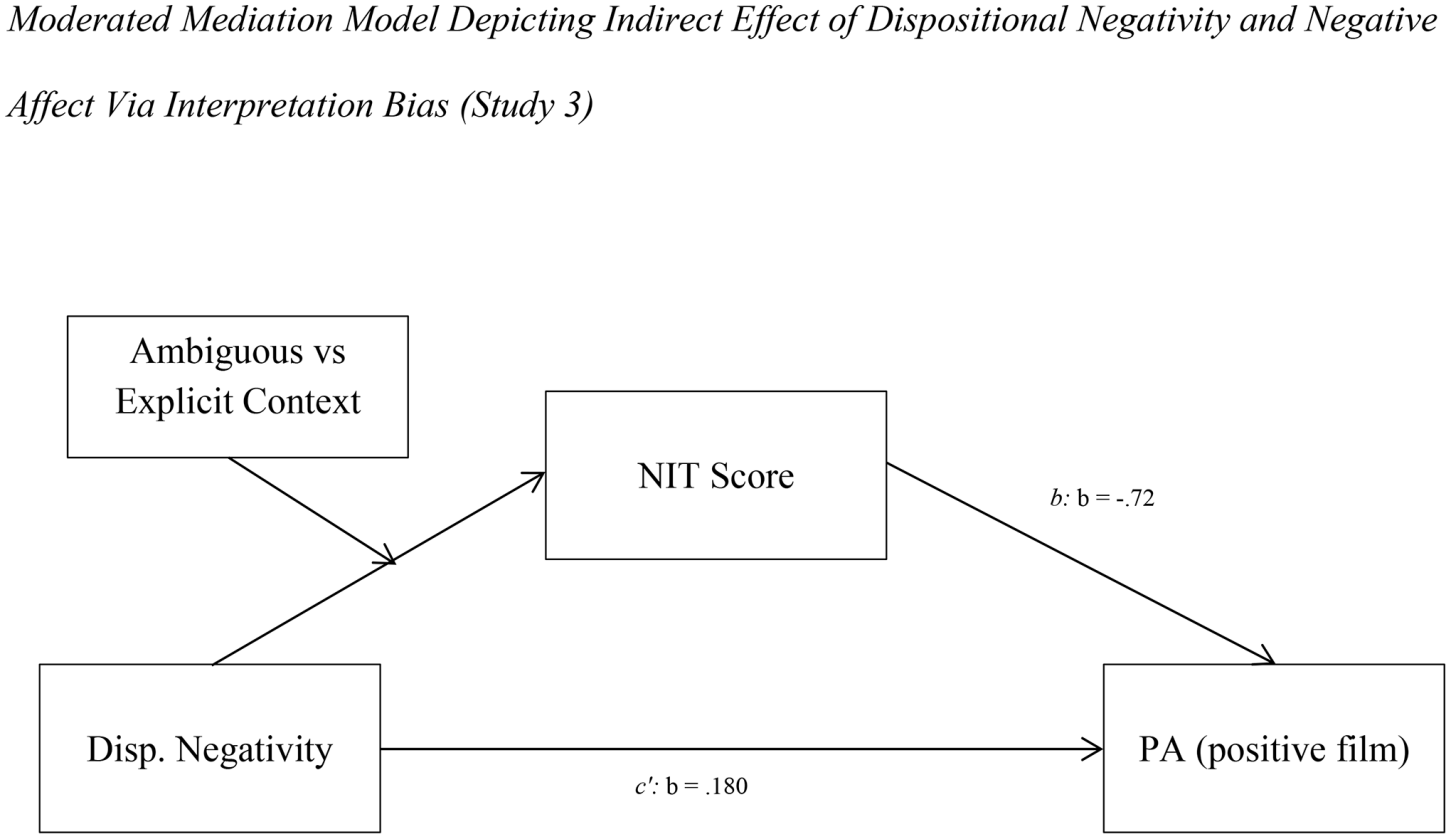
Moderated Mediation Model Depicting Indirect Effect of Dispositional Negativity and Negative Affect Via Interpretation Bias (Study 3).

In our model predicting NA change scores, group was not found to moderate the effect of dispositional negativity on NIT significantly (*b* = .082, SE = .050, t = 1.647, p = .099). The overall moderated mediation model was not supported, according to the index of moderated mediation (Condition = .001 (95% CI = −.008,.013). Since zero was contained within the confidence interval, we did not find evidence for a significant moderating effect of condition on negative affectivity on the indirect effect via NIT. All paths of this moderated-mediation model are depicted in Fig 3.

**Fig 4:**
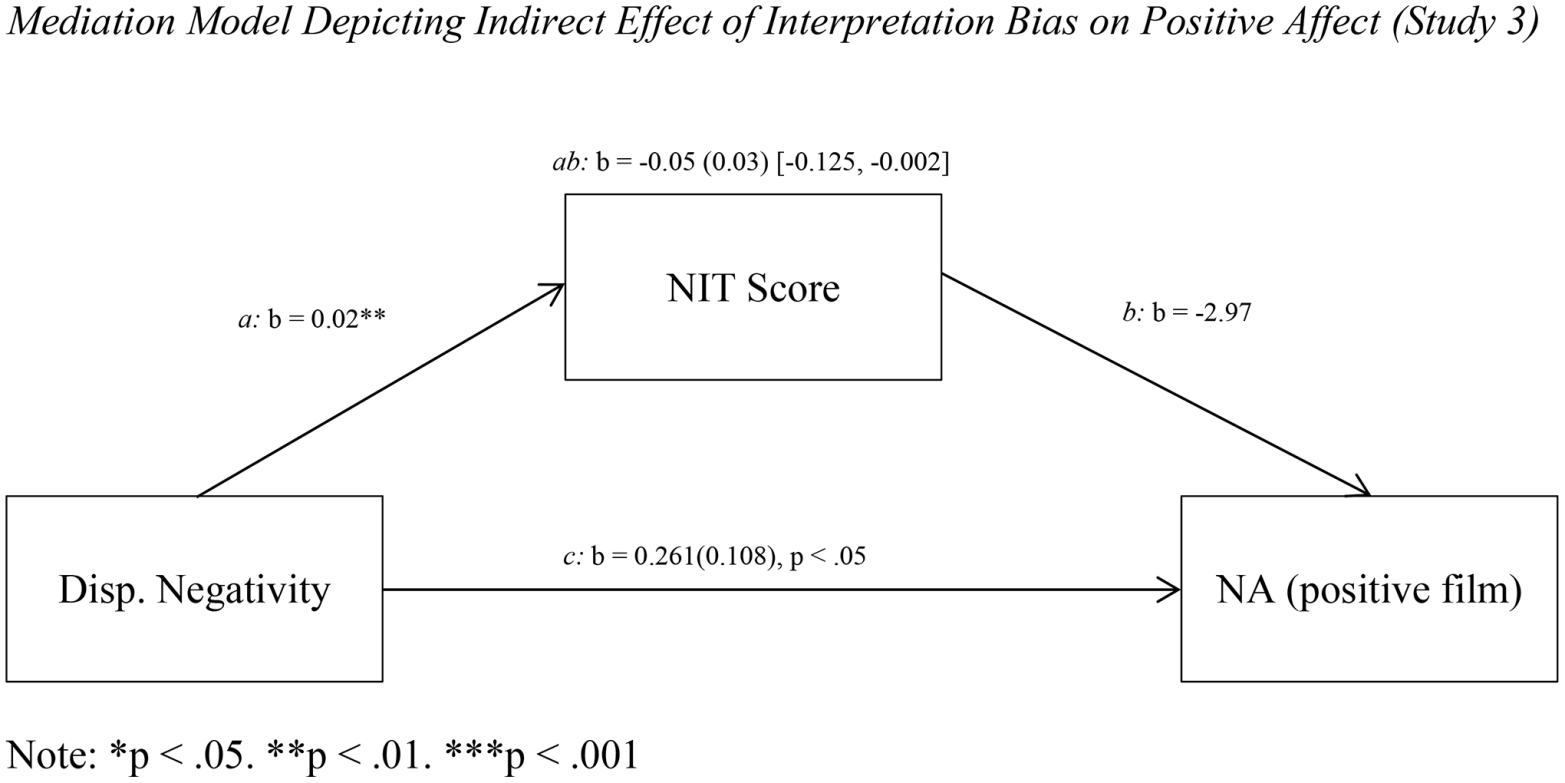
Mediation Model Depicting Indirect Effect of Interpretation Bias on Positive Affect (Study 3).

Because group did not act as moderator in either analyses, we then tested post-hoc, a simple mediation model with reported PA change scores as the outcome variable, and group entered as a covariate. All paths of this mediation model are depicted in Fig 4. The a path, which represents the effect of dispositional negativity on negative interpretation bias, was significant, *b* = .018, SE = .004, *p* = .000. The b path, which represents the effect of negative interpretation bias on PA change scores, was non-significant, *b* = −.855, SE = 1.029, *p* = .406. The c’ path, which describes the direct effect of dispositional negativity on PA change scores, *was* significant, *b* = .175, SE = .078, *p* = .025. However, we found that negative interpretation bias did not significantly mediate the inverse association between dispositional negativity and PA change scores, R^2^ = .053, *b* = −.015, boot SE = 0.09, 95% CI = [−0.011, 0.025].

## Study 3 discussion

In this study, we focused on manipulating the context in which emotion responses were assessed by randomizing participants to view either discrete or mixed/ambiguous positive and negative films. This allowed for a test of group as moderator of the association between NIT scores and emotional responses. However, we did not find significant mediation in either of our two moderated-mediation models.

As extant theory strongly suggests that contextual ambiguity should matter in determining whether interpretation biases will prove influential on affective experiences during positive contexts, these findings are surprising. One explanation for our findings defying expectations may be that the demand of contextual ambiguity was not sufficiently met. A manipulation check did confirm that positive and negative affect were meaningfully different in the mixed and discrete conditions (Table 3). However, this does not necessarily mean that the mixed condition was sufficiently ambiguous to activate cognitive biases. Another explanation, which will be further expanded upon in the discussion to follow, is that these relationships should be modeled using a framework besides moderated-mediation—for example, mediated-moderation.

**Table 3:** Condition manipulation check (Study 3).

Pair	M	SD	t	df	p	Cohen’s d
Discrete Positive Clip PA-Mixed Positive Clip PA	4.00/2.93	1.56/1.59	5.84	292	<.001	1.57
Discrete Positive Clip NA-Mixed Positive Clip NA	1.21/2.00	.49/1.13	−7.69	292	<.001	.87
Discrete Negative Clip PA-Mixed Negative Clip PA	1.73/1.77	.86/.88	−.402	292	.344	.87
Discrete Negative Clip NA-Mixed Negative Clip NA	2.88/2.57	1.05/1.10	2.51	292	<.01	1.07

## General discussion

Together, these three studies aimed to test whether negative interpretation bias might help explain variability in affective responses to positive contexts. We hypothesized that negative interpretation bias would be associated with elevated negative emotional responses and dampened positive emotional responses to a positive film and, further, that negative interpretation bias would mediate the relationship between dispositional negativity and emotional responses to a positive film. In Study 1, we found support for both of our primary hypotheses. Negative interpretation bias was associated with lower positive affect in response to positive films, and we found that it partially mediated the relationship between dispositional negativity and self-reported positive affect. However, this effect was small and when accounting for positive affect ratings collected earlier in the survey, this effect became non-significant. This led us to attempt to replicate that same mediation effect in a second study and to improve upon our methodological design, to attempt to capture true reactivity to positive emotional stimuli.

In Study 2, we embedded the positive film with a neutral and a negative film, to more finely test the uniqueness of the positive context. We also calculated change scores using affect ratings collected just before viewing the positive film. Here, we again tested our same primary hypotheses, though did not find support for either. However, we did find that anhedonic depression symptoms were positively associated with negative interpretation bias, suggesting a potentially larger role for trait-level indicators of positive affective responding than anticipated. Additionally, we found evidence for convergence of the NIT and a well-established task-based measure of negative interpretation bias, the Emotional BADE Task. Yet the NIT was not significantly associated with positive or negative affect in Study 2. This was surprising, leading to questions over whether its predictive validity for emotional reactivity may be more limited than expected, or whether our measurement of emotional reactivity itself (via self-report) could have been suboptimal.

Finally, to understand why significant mediation was found in Study 1 but not in Study 2, we returned to the construct of interest itself and considered the circumstances in which negative interpretation biases tend to manifest. First, these biases are traditionally understood to become most salient in ambiguous contexts [70], where the meaning of a stimulus is uncertain. It may be that when stimuli are explicitly valenced and there is a clear demand, negative interpretation biases exert less influence. For example, the positive film clips used in Studies 1 and 2 were explicitly amusing [48]. Perhaps the explicitly positive nature of these clips made it less likely for negative interpretation biases to be activated. This could explain why the mediation effect found in Study 1 was relatively small and why an effect did not emerge consistently across all studies. For this reason, we conducted Study 3, with the hope of directly testing our hypothesized relationships in an ambiguous context compared to an explicit context. While what we discovered in Study 3 did not support our predictions, theory still suggests that ambiguity may be a necessary prerequisite in order for negative interpretation bias to exert meaningful influence. Additionally, it remains to be seen whether ambiguity is necessary or simply sufficient for the emergence of this effect.

A key takeaway from these three studies is that considering the role of negative interpretation biases in influencing positive emotional responses could be worthwhile, but the relationship may be a very subtle process that is sensitive to method of emotion measurement and even obscured by dispositional affective tendencies. In all three studies, we found evidence suggesting that more stable affective tendencies may play an even more important role in this relationship than had been initially anticipated. In Study 1, we found that covarying earlier reports of positive affect caused the mediation effect to go away. This suggests that trait positive affect could have been driving positive emotional responses in the moment. In Study 2, we found that anhedonic depression symptoms were significantly correlated with dispositional negativity, negative interpretation bias, and mean PA and mean NA in response to the positive film. Similarly, in Study 3 we found that anhedonic depression symptoms were significantly correlated with dispositional negativity, negative interpretation bias, and mean NA in response to the ambiguous positive film. This points to the importance of considering trait indicators of positive affect such as anhedonia in future models and in our broader conceptualization of this relationship.

Surprisingly, there was also inconsistency in the NIT’s association with positive and negative affective responses in Studies 2 and 3. One explanation for this is that different cognitive skills are being employed in the service of completing the homograph-style task and generating self-report affect ratings. Assuming that the NIT is actually sensitive to interpretation bias, this would then mean that the lack of significant association between it and negative and positive affect could be due to distinct differences in how individuals high in these biases tend to rate their own emotional experiences. There is substantial evidence that individuals who display cognitive biases do often also display high levels of alexithymia, or the inability to distinctly characterize emotional experiences [71]. Thus, their ability to reliably report on emotional experiences could be limited, and could help to explain the limited associations that emerged here. If evaluating more objective indicators of affective experience (e.g., discrete facial expressions), the resulting associations may be quite different and suggests an important next step in this line of research.

In sum, our three studies lend some preliminary support to the idea that negative interpretation bias could be one factor contributing to the relationship between dispositional negativity and positive affective responses. However, the inconsistency with which this effect emerged seems to speak to a need to account for other key drivers of emotional responses and the contribution of relevant contextual factors. Specifically, our findings suggest that stable, trait-level indicators of positive affective responding, like anhedonia, could overshadow these biases. However, the argument may also be made that the relationship of interest is a very subtle process that was simply not consistently elicited through this study design. We employed considerable care in successively refining the study design in each of the described investigations. This should set a path for future investigations, where we hope to build on and refine this model. Further investigation is required to gain a clearer understanding of what circumstances lead this bias to matter and how it interacts with well-established, powerful predictors of emotional reactivity.

### Future work

Evidently, there is further work to be done in clarifying the role that interpretation bias plays in influencing affective responses to positive contexts and determining how to best leverage the clear clinical implications of this link. The first of such aims should be to home in on how to best understand this relationship. We found evidence that negative interpretation bias, as measured by the NIT, mediated the relationship between dispositional negativity and affective responses to positive contexts. In Study 3, we tested our model using a moderated-mediation framework. While we did not find significant moderated-mediation in Study 3 and evidence garnered from Study 1 suggests simple mediation might be the most appropriate way to conceptualize this relationship (i.e., negative interpretation bias as a *mechanism*), we should continue to refine our model and evaluate different ways of representing this relationship.

Similarly, extensions of this work should continue to explore the condition of ambiguity. We suspected that the influence of interpretation bias would emerge most clearly in relation to mixed or more ambiguous contexts. However, this was not able to be explicitly shown in Study 3, where we tested mediation in a mixed/ambiguous positive film clip as opposed to a discrete, overtly positive film clip. One explanation for this is that we were unable to adequately simulate true ambiguity in our stimuli. Clearly, future directions should endeavor to better simulate conditions of ambiguity so that this can be properly tested, and the specific circumstances under which cognitive biases are reliably triggered can be better defined.

Our work also demonstrated that there is certainly an effect of trait levels or more stable levels of positive emotional responding that we need to better understand and be sure to include in our conceptualization of this relationship. For example, in both Study 2 and Study 3, anhedonic depression symptoms appeared to be uniquely associated with both dispositional negativity and our interpretation bias task, as well as self-reported affect. Incorporating some kind of trait or dispositional measure of positive affective responding in our model may serve to strengthen it. Additionally, the larger context of the individual could perhaps be a factor to consider as well. In speculating why we found mediation in Study 1 despite the use of more explicitly positive stimuli (i.e., not ambiguous), we might consider the larger context of individuals recruited at that time, namely the COVID-19 pandemic. Such powerful situational factors or contexts may contribute to an individual experiencing a greater level of uncertainty or threat day-to-day. This could very well have helped shape responses to affective stimuli in Study 1, as individuals in that context could be more generally susceptible to the influence of interpretation biases. As such, future work should evaluate the broader context of the individual beyond just reported psychological symptoms, perhaps by using an index of perceived daily stressors or relevant dispositional factors.

Similarly, other alternative mechanisms should be considered. Attentional biases are particularly salient to the central research question. Among individuals high in dispositional negativity, this tendency for attention to orient toward ambiguous materials then results in differences in emotional reactivity [5]. Here, with Studies 1 and 2, we utilized more explicitly positive stimuli (i.e., non-ambiguous stimuli) as a way to potentially parse the influence of interpretation biases from other similar relevant cognitive components of emotional processes, like attentional biases. We would expect that with these non-ambiguous stimuli, attentional biases and other perceptual biases which have more to do with early-stage information processing than with downstream appraisals could be less relevant than interpretation biases. Rather than diminished reactivity as a consequence of over-attending to threatening or potentially threatening information, we attempted to test whether interpretation of positive content as less positive and rewarding may be a mechanism that contributes to diminished positive emotional reactivity. However, it may be that attentional biases play a more prominent role than anticipated and should still be accounted for in some way here.

Another alternative mechanism worth considering is individual variation in emotion regulatory strategy use, particularly positive reappraisal and dampening. Inflexibility in interpretation has been convincingly associated with habitual use of particular emotion regulation strategies that act on positive emotion, like positive reappraisal and dampening (Everaert et al., 2020) [32]. Given the clear linkage of these strategies to either positively or negatively enhancing the intensity and duration of positive emotion, assessing what individuals are doing in the moment to regulate emotion is worthwhile. It could very well be that the effect of negative interpretation bias on positive emotional reactivity is also tied to distinct differences in emotion regulatory strategy use.

Finally, further work should continue to test the utility of the NIT. In Study 2, we found that the NIT showed convergent validity when compared to the Emotional BADE Task, a well-established measure of negative interpretation bias. Moreover, the NIT and the Emotional BADE Task appeared complementary but not redundant. Future work could further test this novel task, to parse what processes or subprocesses it may be tapping into compared to other interpretation bias tasks. It should be noted that there is significant variety in paradigms used to measure negative interpretation bias. One recent review identified 45 different such paradigms [72]. These paradigms could be broadly categorized as utilizing either single ambiguous words, ambiguous images, or ambiguous scenarios, and each has their respective strengths and limitations though all have in common some presentation of ambiguous stimuli and some assessment of threat or emotional interpretations made. A relatively short, easily administered task that can assess negative interpretation biases provides a convenient tool for exploring these and related research questions.

### Clinical implications

Another compelling reason to continue investigating the influence of negative interpretation biases in positive contexts is the clear clinical application of this work. Better understanding how these biases contribute to individual differences in positive affective experiences may prove especially beneficial. Diminished responding to positive emotional experiences is predictive of increased risk of psychiatric disorders broadly [73] but also predicts poorer course and greater symptom severity in individuals with depressive disorders, as well as a lower likelihood of benefiting from antidepressant medications [74,75]. Identifying factors that predict diminished responses to positive contexts is a critical first step to addressing and ameliorating these deficits.

Furthermore, elucidating the relationship of negative interpretation biases to affective processes could help inform the design of existing protocols for modifying cognitive biases [76]. Researchers have noted that while more cognitive bias modification (CBM) treatment work has focused on the effect of CBM on attention biases, CBM treatments appear to actually be more effective in modifying interpretation biases [77]. This does suggest that interpretation biases may operate differently from other cognitive biases, such as attention biases, and they are perhaps more susceptible to modification. Hence, negative interpretation biases may be a more appealing target for intervention. To go one step further, our investigation suggests that treatments which modify negative interpretation biases could have an impact on not just affective responses to negative contexts but also affective responses to positive contexts, making this an even more promising avenue.

The link between interpretation biases and positive affective experiences may also have broader implications for other types of interventions beyond just CBM. For example, this relationship could be helpful in improving treatments that are tailored to enhancing positive affect and addressing diminished sensitivity to reward [78,63]. Even greater gains than presently seen could be made if such interventions were to leverage bias modification in the service of addressing low positive affect. Incorporating interpretation biases into these and other modalities which focus on low positive affect may be hugely beneficial to treatment outcomes, particularly for individuals who experience severe and recurrent depressive symptomology commonly associated with low positive affect.

While interpretation biases certainly present an appealing treatment target due to occurring more downstream and thus being more easily acted-upon, the exact role they may play in driving diminished positive emotional reactivity is still unclear. Clinical applicability should be considered further but also within the broader context of constructs shown to be strongly predictive of positive emotional reactivity, like trait-level positive affect and anhedonia. However, a key limitation of these trait-level factors is that they cannot show use the processes at play at the moment level. It may be that interpretation biases can help to reveal the mechanisms which drive change in this manner.

### Limitations

While this investigation benefits from how each subsequent study improved upon the limitations of the previous, there are general limitations which ought to be acknowledged. Across all three studies, our sample was comprised of college students from a large, Midwestern public university. This may limit the generalizability of our findings to the population as a whole. Testing this effect in samples that are more diverse in age, gender, and racial/ethnic identity, as well as outside of the college context, should be an eventual goal.

One limitation specific to Study 1 was that our data analyses were conducted using an existing dataset, and that these data were originally collected for a different purpose. This resulted in the inability to make certain study design decisions relevant to our research questions. For example, participants in Study 1 only saw positive film clips, thus there was no real comparison or way to individuate that positive context in Study 1. Importantly, the lack of proper pre-film affect ratings also resulted in the calculation of scores which may more closely reflect state affect rather than true reactivity. This was rectified in Study 2’s design, as we included neutral and negative film clips alongside the positive film clip, and were able to calculate change scores using pre-film affect ratings. However, this difference in outcome measurement could be one reason why significant mediation is not found in Study 2 and Study 3. Furthermore, the emotionally-evocative clips viewed before the positive film clips in Studies 2 and 3 were negative for some participants and positive for others. Conducting a test of order effects in Study 2 did not reveal a meaningful difference between participants who saw a negative film clip before the positive film clip (R2 = .015, b = −.003, boot SE = 0.007, 95% CI = [−0.022, 0.081]). That is, significant mediation did not emerge when accounting for order of film clip presentation in Study 2. Regardless, this is another example of a difference in study design which should be noted as a potential limitation and certainly considered when evaluating the results across all three studies.

The assessment of negative interpretation bias could also be further improved upon. While the NIT displayed good reliability and convergent validity across studies, it was not always significantly associated with positive or negative affect, as in Studies 2 and 3, making its predictive validity limited. The inconsistency of this association could also be owed to the measurement of emotional experiences. For example, it may be that the NIT is sensitive to interpretation bias but that self-reported affect ratings may not be the best way of evaluating how these individuals are actually reacting. We know that individuals who display interpretation biases often also display high levels of alexithymia [Luminet et al., 2024]. It may be that their ability to reliably report emotional experiences in this manner is limited and that we could see different patterns emerging if we were to look at more objective indicators of affective experience.

Another limitation that should be noted is the smaller sample size of Study 2, compared to Study 1. This could have resulted in our analyses being underpowered, which is perhaps why we did not replicate our Study 1 finding in Study 2. Regardless, a larger sample size is desirable because it reduces the chances of a Type I or Type II error, hence this was remedied in Study 3.

Finally, it may be prudent to use other kinds of emotionally-evocative stimuli in future work, as, the challenge of identifying truly neutral emotional stimuli is also quite important. It could be argued that the “mixed” stimuli used in Study 3 were in fact interpreted as a mash-up of positive and negative information rather than emotionally ambiguous. Addressing such issues in future work is in line with our intentions to further map in just what contexts interpretation biases may exert the most influence over affective responses.

### Conclusions

In sum, this work provides preliminary evidence that negative interpretation biases may influence variability in affective responses to positive contexts. This potential link holds significance for both clinical and nonclinical populations, as positive emotional experiences are a central feature in psychological health and wellbeing [79]. Better understanding this relationship could help to explain one potential source of low or diminished responses to positive contexts. Moreover, it could lead to recommendations for cultivating wellbeing and greater or more frequent positive affect by intentionally targeting these biases, as has been done with cognitive bias modification training intended to reduce negative affect ([22,76,80,81]). However, as evidenced by this investigation, further exploration is needed to clarify the necessary conditions under which negative interpretation biases shape emotional experiences. This is especially true given the relative weight of trait-level indicators of positive affect like anhedonia relative to other predictors. Future work should aim to establish precisely under what conditions interpretation biases hold sway over affective processes, as well as directly test these biases in contexts that are even more relevant to clinical questions. As well as potentially providing a window into actual mechanisms which are relevant to changes in emotional experience at the moment level, these biases are, importantly accessible. Thus, they present a distinct opportunity for clinical intervention due to being much more targetable and changeable than more static, trait-level features.
